# Correlates of post-partum intra-uterine copper-T devices (PPIUCD) acceptance and retention: an observational study from North India

**DOI:** 10.1186/s40834-023-00222-2

**Published:** 2023-03-28

**Authors:** Sneha Gupta, Romi Bansal, Harbhajan Kaur Shergill, Pradeep Sharma, Priyanka Garg

**Affiliations:** 1 Department of Obstetrics and Gynaecology, Adesh Institute of Medical Sciences and Research, 151001 Bathinda, India; 2 Department of Obstetrics and Gynaecology, All India Institute of Medical Sciences, 151001 Bathinda, Punjab India

**Keywords:** Long-acting reversible contraception, Postpartum period, Acceptance, Retention, Counseling

## Abstract

**Background:**

Postpartum intrauterine contraceptives device (PPIUCD) offers an effective means of providing contraceptive services to women in countries with high rates of unmet needs for family planning services. However, scientific literature estimating the long-term retention rates is scarce. We estimate the factors affecting acceptance and retention of PPIUCD and explore the risk factors against PPIUCD Discontinuation at six months”.

**Material and method:**

: This prospective observational study was conducted between 2018 and 20 at a tertiary care institute in North India. PPIUCD was inserted following a detailed counseling session and consent. The women were followed up for six months. Bivariate analysis was done to depict the association between socio-demographic characteristics and acceptance. Logistic regression, cox regression, and Kaplan Meier analysis were applied to explore factors affecting acceptance and retention of PPIUCD.

**Results:**

Of the 300 women counseled for PPIUCD, 60% accepted them. The majority of these women were between 25 and 30 years (40.6%), primigravida (61.7%), educated (86.1%), and from urban areas (61.7%). Retention rates at six months were about 65.6%, while 13.9% and 5.6% were either removed or expelled. Women declined PPIUCD due to refusal by spouses, partial knowledge, inclination towards other methods, non-willingness, religious beliefs, and fear of pain and heavy bleeding. Adjusted logistic regression depicted that higher education, housewife status, lower-middle and richest SES, Hinduism, and counseling in early pregnancy promoted acceptance of PPIUCD. The most common reasons for removal were AUB, infection, and family pressure (23.1%). Adjusted hazard ratio depicted religion other than Hinduism, counseling in late stages of pregnancy, and normal vaginal delivery were significant predictors for early removal or expulsion. While education, higher socio-economic status favoured retention.

**Conclusion:**

PPIUCD is a safe, highly effective, low-cost, long-acting, and feasible method of contraception. Skill enhancement of healthcare personnel for insertion techniques, adequate antenatal counseling, and advocacy of PPIUCD can help increase the acceptance of PPIUCD.

## Introduction

India has been experiencing exponential growth in population in recent decades, mainly driven by progress in the socio-economic and medical fields. This burgeoning population exerts colossal pressure on the already constrained resources, which have proved limited over time. As per the fourth round of the National Family Health Survey- an Indian counterpart for the Demographic Health Survey- Intrauterine devices have a meager contribution of just 1.5% among all the methods of contraception practiced for family planning. However, IUCD services are offered free of cost at all government facilities [1]. This is mainly attributed to the low level of knowledge, myths, and misconceptions, particularly for copper T, which has resulted in its low utilization and discontinuation [2].

Consecutive pregnancies occurring within 24 months of a previous birth have a higher risk of adverse outcomes [3, 4]. This calls for reliable and effective long-term contraception in the postpartum period. IUCD is a highly recommended method of contraception due to its safety, efficacy, coitus independence, rapidly reversible, and long-acting nature with relatively few side effects [5]. Also, women are highly motivated during the postpartum period and have minimal need for additional hospital visits [6]. Insertion of IUCD in the immediate postpartum period (PPIUCD) is a technique of insertion of IUCD within 48 h of vaginal delivery or cesarean section after removal of the placenta. The copper ions released from the IUCD offer long-term contraception by interfering with the ability of sperm to survive and ascend the fallopian tube where fertilization occurs. It also stimulates a sterile foreign body reaction in the endometrium potentiated by copper [7].

In India, PPIUCD is still emerging as a new contraceptive choice where delivery may be the only time a healthy woman comes in contact with health care personnel. It has been observed that the expulsion rate of PPIUCD varies according to the clinician’s skill. In addition, follow-up care of the PPIUCD is critical to ensure client satisfaction and the continuation of the accepted method [8]. Limited studies have been conducted so far in India about the safety, follow-up data on complications, decision-making, perception, and satisfaction among the women who accepted PPIUCDs [7, 9–11]. In this context, we did the present study to estimate the factors affecting acceptance and retention of PPIUCD and explore the risk factors against PPIUCD discontinuation at six months” in women undergoing delivery in our institution.

## Methods

### Study area and period

The study was conducted between 2018 and 20 in a tertiary care teaching health facility in the Malwa region of Punjab, India’s northern state. It is a private facility where family planning, Antenatal care (ANC), and delivery services are provided at a nominal charge. It serves as a referral center for complicated ante-natal cases by offering state-of-the-art blood banks, operation theatre, and intensive care for the mother and child. Modern contraceptive methods (injectables, pills, implants, male condoms) are offered to clients in need after proper counseling and assessing their medical eligibility using the Medical Eligibility Criteria for Contraceptive Use (MEC) wheel as recommended by the World Health Organization.

### Study design

A facility-based prospective observational study design was employed.

### Study participants

Pregnant women between 28 and 42 weeks of gestation willing to use IUCD for postpartum contraception within 48 h of delivery were included in the study after explaining the purpose of the research and taking informed written consent to participate in the study. Pregnant women who did not fulfill World Health Organization medical eligibility criteria for IUCD insertion like those with HIV, antepartum hemorrhage, fever during labor and delivery, delivery at less than 28 weeks and with PROM more than 18 h, who had a previous history of genital tuberculosis, known allergy to copper, history of uterine abnormalities and not willing to participate were excluded from the study.

The Counseling and process of having consent were done per the guidelines by the Government of India [12]. During the process of giving counseling and obtaining informed consent from the participants, they were explicitly told about the possible benefits of using the PPIUCD, potential side effects and complications, the process of getting the PPIUCD removed, precautions to be taken, how to observe the PPIUCD and other available options of contraception in the postpartum period. Specifically, we ensured that she knows that menstrual changes are a common side effect among PPIUCD users and that the PPIUCD does not protect against STIs. Also, we described the medical assessment required before PPIUCD insertion and the procedures for PPIUCD insertion and removal. Concurrently, we encouraged her to ask questions throughout the process and provided any other additional information and reassurance as needed.

### Sample size and sampling

A sample size of 289 was calculated using the single population proportion formula after considering the overall acceptance of PPIUCD to be around 25%[13], with a 95% confidence interval and a margin of error of 5%. We included a total of 300 participants in the study. Eligible pregnant women were recruited from the OPD or emergency labor room using a systematic random sampling technique.

### Data collection

The structured and pre-tested questionnaire was prepared first in English from peer-reviewed articles and then translated into Hindi and Punjabi (local languages), using the standard WHO methodology for questionnaire translation. The tool consisted of three sections: Part A included questions to collect information regarding the socio-demographic factors (age, education, occupation, socio-economic status, religion, residence) and obstetric history (parity, timing of PPIUCD insertion, client perception of pain during and after PPIUCD insertion and time since last childbirth); while part B had questions about the acceptance of PPIUCD, decision making about PPIUCD ( Period of counseling, decision making in PPIUCD as family planning method, reasons for acceptance and declining of PPIUCD, Time of the decision taken to choose PPIUCD as family planning method), and Part C collected data from the follow-up visits (acceptance of PPIUCD, side-effects, reason for removal and satisfaction). The study tool was pilot tested in 30 pregnant women and incorporated necessary changes in the final version. The authors collected data after ensuring the study subjects’ confidentiality and privacy, maintaining a non-judgemental attitude to minimize bias.

After taking universal precautions, a procedure was carried out on patients who consented to participate in the study. In women who had a normal vaginal delivery, after the expulsion of the placenta, the IUCD was held in suitably long forceps without a lock (Kelly’s forceps). The instrument was taken to the uterine fundus, and the IUCD was released. While in a cesarean section, the IUCD was introduced through the uterine incision after removal of the placenta and placed at the uterine fundus. This was done manually or using artery forceps, and the strings were directed towards the os.

Women who accepted PPIUCD insertion were advised to follow up routinely after six weeks, three months, and six months on an outpatient basis. On follow-up visits, the position of IUCD was verified by per speculum and vaginal examination. If the participant did not feel the threads, pelvic ultrasound or radiography of the pelvis was done. The findings on their follow-up visit like expulsion, reasons for removal, continuation rate, loss to follow up, and any complications like menstrual problems, infection/discharge per-vaginum, pyrexia, abdominal pain or backache, lost or missing thread, perforation, and pregnancy were noted.

### Data analysis

Data was entered in the Microsoft Excel sheet and checked for completeness and inconsistencies by the principal investigator. It was analyzed using SPSS version 21( IBM SPSS Statistics for Windows, Version 21.0. Armonk, NY: IBM Corp). We conducted a bivariate analysis to assess any association between independent variables (the socio-demographic characteristics and counseling process) and the dependent variable (PPIUCD acceptance). Further, we did a multivariable logistic regression analysis to explore factors affecting the acceptance of the PPIUCD. At the same time, a Cox proportional hazard model was used to estimate hazard ratios (HRs) of variables associated with discontinuation. Independent variables with a significant association (p < 0.2) in the bivariable analysis were entered into the multivariable analysis. The final model declared a significant association at a p < 0.05. The results were presented in tables with adjusted odds ratio (AOR) and the corresponding 95% confidence interval. Kaplan-Meier survival function was used to estimate the continuation rates for the PPIUCD at six months after insertion.

### Ethical considerations

Ethical approval was obtained from the research review committee of Adesh Institute of Medical Sciences and Research, Bathinda, Punjab (AU/EC/FM/133/ 2018). We obtained written informed consent from each study participant. We also ensured the confidentiality of the information to all participants throughout the study, and the data was made anonymous for analysis. Withdrawal from the study at any point was assured to all the participants.

## Results

Of the 300 women counseled for PPIUCD, 60% accepted using it as a contraceptive method in their postpartum period. **(**Table 1**)** Acceptance of PPIUCD was significantly higher (p-value < 0.05) in educated women, homemakers, higher socio-economic status, following Sikhism and Hinduism, rural areas of residence, positive history of family planning usage in the past, and even the types of methods used in the past. However, the participant’s age, gravidity, and time since the last childbirth did not affect the chances of accepting PPIUCD. Further, we assessed the effect of counseling on the prospects of acceptance **(**Table 2**).** It was seen that the acceptance was significantly higher when counseling was provided in the ante-natal period when the respondent was the sole decision-maker and those who ended up having a cesarean section. All of the respondents who were counseled (n = 180) and finally accepted PPIUCD reported satisfaction with the counseling services. The most common reason for PPIUCD acceptance in the present study was its long lifetime period (42.2%), non-hormonal action (16.1%), non-interference with breastfeeding (15.6%) as seen with hormonal pills, the safety of usage (10.6%), need for fewer follow-ups (8.3%) and reversibility of fertility after removal (7.2%). However, women who declined (n = 120) PPIUCD cited reasons of either refusal by their husbands (23.33%), concern about the partial/incomplete knowledge (18.3%), preference for other methods (17.5%), non-willingness to adopt any contraception immediately (15.8%), religious beliefs (15%) and due to fear of pain and heavy bleeding after insertion (10%). Nevertheless, in our study, 67.2% of participants perceived no pain at the time of PPIUCD insertion (immediately following the delivery), while only 8.3% admitted it to be very painful (data not tabulated).

**Table 1:** Association between Socio-demographic variables and PPIUCD acceptance among the study participants

	PPIUCD acceptance			p-value
	No	Yes	Total	
**Total**	120(40)	180(60)	300(100)	
**Age in years**				0.592
< 26 years	17(39.5)	26(60.5)	43(100)	
26–35 years	95(41.1)	136(58.9)	231(100)	
> 35 years	8(30.8)	18(69.2)	26(100)	
**Education**				< 0.001
Literate	46(22.9)	155(77.1)	201(100)	
Illiterate	74(74.7)	25(25.3)	99(100)	
**Occupation**				< 0.001
Employed	15(34.9)	28(65.1)	43(100)	
Housewife	90(39.8)	136(60.2)	226(100)	
Laborers	0	16(100)	16(100)	
Others	15(100)	0	15(100)	
**Socio-economic status**				0.001
Poorest	32(58.2)	23(41.8)	55(100)	
Lower middle	34(36.6)	59(63.4)	93(100)	
Upper middle	37(46.3)	43(53.8)	80(100)	
Richest	17(23.6)	55(76.4)	72(100)	
**Religion**				< 0.001
Hindu	73(39)	114(61)	187(100)	
Muslim	27(67.5)	13(32.5)	40(100)	
Christian	15(41.7)	21(58.3)	36(100)	
Sikhs	5(13.5)	32(86.5)	37(100)	
**Area of residence**				0.002
Rural	26(27.4)	69(72.6)	95(100)	
Urban	94(45.9)	111(54.1)	205(100)	
**Family Planning usage in past**				0.003
No	42(77.8)	12(22.2)	54(100)	
Yes	108(43.9)	138(56.1)	246(100)	
**Type of Family planning used in the past**				0.006
Condom	35(42.2)	48(57.8)	83(100)	
Pills	31(50)	31(50)	62(100)	
Injectable	18(43.9)	23(56.1)	41(100)	
Others	24(46.2)	28(53.8)	52(100)	
IUCD	0	8(100)	8(100)	
No method	42(77.8)	12(22.2)	54(100)	
**Gravid**				0.661
Multigravida	43(38.4)	69(61.6)	112(100)	
Primigravida	77(41)	111(59)	188(100)	
**Time since last childbirth**				0.086
< 2 years	17(30.4)	39(69.6)	56(100)	
2–3 years	23(52.3)	21(47.7)	44(100)	
> 3 years	3(23.1)	10(76.9)	13(100)	
Not applicable	77(41.2)	110(58.8)	187(100)	

**Table 2:** Association between the process of counseling and the acceptance of PPIUCD among the study participants

	PPIUCD acceptance			p-value
	No	Yes	Total	
**Total**	120(40)	180(60)	300(100)	
**Period of counseling**				< 0.001
Antenatal	39(26.9)	106(73.1)	145(100)	
Immediate postpartum	38(42.7)	51(57.3)	89(100)	
Intrapartum (Early Labour)	43(65.2)	23(34.8)	66(100)	
**Satisfied with counseling**				< 0.001
No	75(100)	0	75(100)	
Yes	45 (20)	180(80)	225(100)	
**Decision Taken by**				< 0.001
Self	40(30.1)	63(69.9)	59(100)	
Husband	60(48.8)	63(51.2)	63(100)	
mother	5(35.7)	9(64.3)	43(100)	
Mother-in-law	15(50.0)	15(50.0)	15(100)	
**Mode of the delivery**				< 0.001
Cesarean section	70 (40.7)	102(59.3)	172(100)	
Normal vaginal	42 (48.8)	44(51.2)	86(100)	
Instrumental	8 (19.1)	34(80.9)	42(100)	

*Figures in parenthesis depicts percentages*

**Fig. 1:**
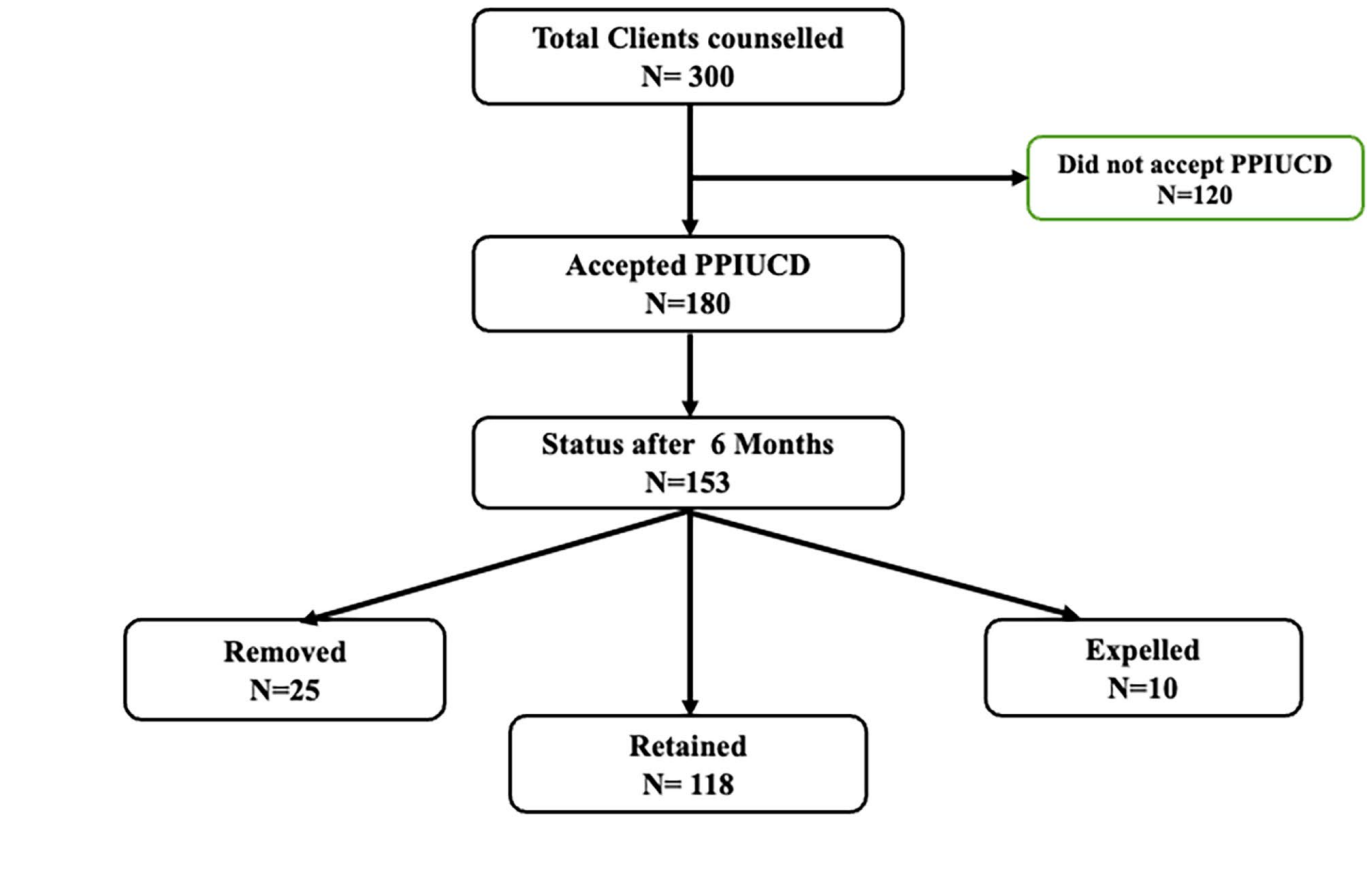
Status of PPIUCD by the end of 6 months among the participants visiting a tertiary care hospital in the Malwa region of Punjab

By the end of 3 months, the proportion of respondents who retained their PPICUD was 65% and remained the same even at six months. Among those non-compliant, 6.7% and 3.3% of respondents got their PPICUD removed by the end of six weeks and six months, respectively. In 3.3% and 5.6% of cases, IUCD got expelled spontaneously, while 6.7% and 15% could not be contacted and were lost to follow-up **(**Table 3; Fig. 1**)**. We also explored the reasons for removing PPIUCD from 12 to 13 women who got Cu T removed at six weeks and three months. At six weeks, the main reasons included AUB (66.67%) and infection (16.67%), while at three months, the reasons included AUB (53.84%), and family pressure (23.1%). We also recorded the complications following PPIUCD insertion at the three follow-up visits. At six weeks, 62 women reported complications which mainly included AUB (10.7%), missing threads (9.5%), and infection/per-vaginal discharge (8.3%). At the end of 3 months, the most common complication was AUB (10.2%), while infection and per-vaginal discharge (3.8%) were most commonly reported at six months.

**Table 3:** Status of PPIUCD at long-term follow-up visits (n = 180) among the study participants who accepted the PPIUCD as a method of contraception

	Follow up at		
	Six week	Three months	Six months
**Status of PPIUCD**			
Retained	150(83.3)	118(65.6)	118(65.6)
Removal	12(6.7)	25(13.9)	25(13.9)
Expulsion	6(3.3)	10(5.6)	10(5.6)
Loss to follow up	12(6.7)	27(15)	27(15)
**Reasons of removal**			
Abnormal Uterine Bleeding	8(66.7)	7(53.84)	0
Missing thread	1(8.3)	1(7.7)	0
Family pressure	1(8.3)	3(23.1)	0
Infection	2(16.7)	2(15.4)	0
**Complications**			
Infection/Discharge P/v	14(8.33)	8(5.44)	4(3.39)
Backache/Abdominal pain	10(5.95)	7(4.8)	2(1.7)
AUB	18(10.71)	15(10.20)	3(2.54)
Missing threads	16(9.523)	1(0.7)	0
Perforation	0	0	0
Pyrexia	4(2.4)	0	0

*Figures in parenthesis depicts percentages*

**Fig. 2:**
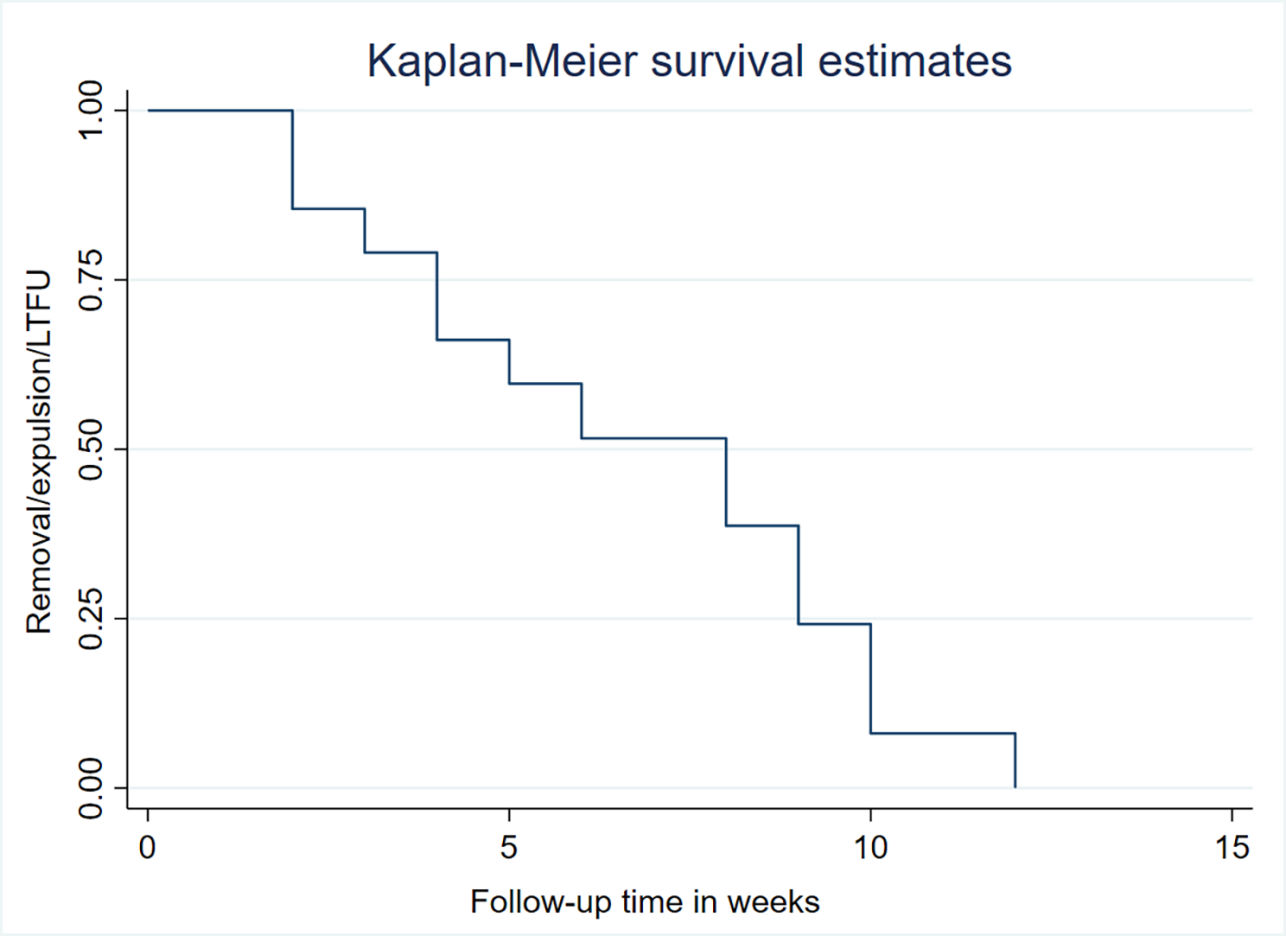
Kaplan-Meier survival estimate of PPIUCD continuation at six months

Further, unadjusted binary logistic regression depicted that the most common factors for acceptance of PPIUCD included age between 26 and 35 years, education, employed or housewife, from the lower middle or richest SES, Hindu and Muslim religion, urban residence, primigravida, and counseling in the immediate postpartum period **(**Table 4**)**. However, adjusted logistic regression depicted education, housewife, lower-middle and richest SES, Hinduism, and counseling in the antenatal and immediate postpartum period as final predictors of acceptance of PPIUCD during counseling sessions. Then, a bivariate analysis of the participants who received a PPIUCD with different socio-demographic variables depicted that type of occupation, religion, and time of counseling for PPIUCD affected retention significantly. Cox hazard regression analysis illustrated that the chances of the event (removal and expulsion) happening were significantly higher in women following religion other than Hinduism, those who had received counseling for the first time during the intrapartum period or had undergone normal vaginal delivery, while education, unorganized labor class, higher socio-economic status emerged as protective factors against the removal or expulsion. **(**Table 5**).** Figure 2 shows Kaplan-Meier survival estimates for PPIUCD retention at the end of six months.

**Table 4:** Logistic regression analysis to describe the correlates of acceptance of PPIUCD during the counseling session

Variable	Unadjusted odds ratio (95% CI)	p-value	Adjusted odds ratio (95% CI)	p-value
**Age in years**				
< 26 years	**Ref**		**Ref**	
26–35 years	1.4 (1.1–1.8)	0.007	1.3 (0.5–3.2)	0.533
> 35 years	2.2 (1.0-5.2)	0.056	3.2 (0.8–12.9)	0.101
**Education**				
No	**Ref**		**Ref**	
educated	3.4 (2.4–4.7)	0.000	14.8 (6.9–31.6)	0.000
**Occupation**				
Employed	1.9 (0.9–3.5)	0.05	0.7 (0.3–1.8)	0.494
Housewife	1.5 (1.1–1.9)	0.002	2.1 (1.7–3.2)	0.040
Labourers	1.6(0.0–1.0)	0.998	1.9 (1.2–2.5)	0.041
Others	**Ref**		**Ref**	
**Socio-economic status**				
Poorest	**Ref**		**Ref**	
Lower middle	1.7 (1.1–2.6)	0.100	7.3 (2.7–19.7)	0.000
Upper middle	1.2 (0.7–1.8)	0.503	2.4(0.9–6.2)	0.066
Richest	3.2 (1.9–5.6)	0.000	5.5 (1.9–15.3)	0.001
**Religion**				
Sikhs	**Ref**		**Ref**	
Muslim	1.5 (1.2–2.1)	0.003	1.3(0.5–3.8)	0.525
Christian	0.5 (0.2–0.9)	0.030	0.3 (0.9–1.2)	0.091
Hindu	6.4 (2.5–16.4)	0.000	4.2 (1.2–18.4)	0.049
**Area of residence**				
Rural	**Ref**		**Ref**	
Urban	2.6 (1.7–4.2)	0.000	1.6 (0.7–3.6)	0.204
**Family Planning usage in past**				
no	**Ref**		**Ref**	
yes	3.5 (1.8–6.6)	0.000	2.9 (1.2–7.3)	0.019
**Gravid**				
Multigravida	**Ref**		**Ref**	
Primigravida	1.6 (1.1–2.3)	0.015	1.3 (0.6–2.6)	0.461
**Period of counseling**				
Antenatal	1.3 (0.8-2.0)	0.170	5.1 (2.1–12.6)	0.000
Immediate postpartum	0.5 (0.3–0.9)	0.015	3.7 (1.4–9.4)	0.006
Intrapartum	**Ref**		**Ref**	

*Ref: Reference values*

**Table 5:** Adjusted Hazard Ratios (aHR) using Cox-regression analysis for exploring the risk factors against PPIUCD Discontinuation at six months

	Total	Retention at six months	P-value	Adjusted HR (95% CI)	P-value
**Total**	180(100)	118(65.6)			
**Age of the women**			0.386		
< 26 years	26(100)	15(57.7)		**Ref.**	
26–35 years	136(100)	89(65.4)		1.1 (0.5–1.9)	0.908
> 35 years	18(100)	14(77.8)	0.860	0.5 (0.2–1.8)	0.333
**Education**					
Not Educated	25(100)	16(64)		**Ref.**	
Educated	155(100)	102(65.8)		0.6 (0.4–0.8)	< 0.01
**Occupation**			0.026		
Housewives	136(100)	83(61)		**Ref.**	
Employed	28(100)	20(71.4)		0.6 (0.3–1.3)	0.229
Others	16(100)	15(93.8)		0.1 (0.1–0.8)	0.026
**Socio-economic Status**			0.305		
Poorest	23(100)	14(60.9)		**Ref.**	
Lower middle	59(100)	35(59.3)		0.9 (0.4–2.1)	0.945
Upper middle	43(100)	33(76.7)		0.4 (0.2–0.9)	0.048
Richest	55(100)	36(65.5)		0.7 (0.3–1.5)	0.383
**Religion**			< 0.01		
Hindu	114(100)	80(70.2)		**Ref.**	
Non-Hindu	66(100)	38(57.6)		1.7 (1.1–2.7)	0.049
**Residence**			0.805		
Rural	69(100)	46(66.7)		**Ref.**	
Urban	111(100)	72(64.9)		0.8 (0.5–1.4)	0.410
**Family Planning methods usage in past**			0.199		
No	42(100)	31(73.8)		**Ref.**	
Yes	138(100)	87(63)		1.6 (0.8-3.0)	0.142
**Period of counselling**			0.004		
Antenatal	106(100)	70(66)		**Ref.**	
Intrapartum	23(100)	11(47.8)		2.0 (1.4–3.9)	0.035
Immediate postpartum	51(100)	37(72.5)		0.8 (0.4–1.7)	0.649
**Time since Last Childbirth**			0.497		
NA (Primigravida)	110(100)	70(63.6)		**Ref.**	
Up to 3 years	70(100)	48(68.6)		0.8 (0.4–1.7)	0.649
**Mode delivery**			0.329		
Caesarean Section	102(100)	71(69.6)		**Ref.**	
Instrumental (assisted)	34(100)	19(55.9)		1.2 (0.7–2.3)	0.417
Normal vaginal delivery	44(100)	28(63.6)		1.7 (1.2–3.3)	0.045

*Ref: Reference values*

## Discussion

We highlight the effect of adequate counseling services on the long-term retention of PPIUCD and thus harness their actual potential as a practical family planning method. The revival of PPIUCD by the Ministry of Health and Family Welfare, Government of India, with technical assistance from Jhpiego in 2010, led to conscious efforts to provide the benefits of this long-term reversible postpartum contraception [14]. In our study, 60% of women accepted PPIUCD as a method of contraception during their postpartum period. This is higher than the acceptance observed by Agarwal N al et al. (41.1%) and Gautam R et al. (21.8%) [11, 15]. The origin of these studies was from the low-performing states of India in terms of Family planning. To add, in our institute, Cu T 380 A is available free of cost with support from the Government of India. Therefore, numerous advantages supplemented by no-cost contraceptive makes pregnant women more receptive to PPIUCD. Many socio-demographic factors are responsible for higher acceptance and are discussed in the subsequent parts of the manuscript.

We observed that the acceptance of PPIUCD as a long-term family planning method is significantly affected by education, SES, religion, and appropriate counseling, especially in the antenatal period. Similar results were seen in a study by Rajni Gautam et al., Kanhere AV et al., and Pandher et al. [15–17]. Nearly 77% of our acceptors were educated women, similar to other studies [11, 17]. Most of them belonged to urban areas, which was in concurrence with the study conducted by Patel J et al. (93.14%) and Mule VD et al. (62.5%)[18, 19]. This reveals that women’s education status and urban residence significantly influence the acceptance of PPIUCD. This also highlights the need for targeted interventions for better acceptance among less-educated women and those in rural areas where healthcare services are relatively deficient and inaccessible. We observed that acceptance for PPIUCD was highest in the wealthiest economic group. However, Agarwal N et al. noted higher acceptance in those with lower socioeconomic status [11]. The difference needs further evaluation to account for undue disparities based on SES. We also observed that the acceptance was not affected by the parity of women. However, primiparous women were more inclined towards the spacing method, while multipara women were interested in the permanent form of contraception. Similar results were seen in the study by Garuda L et al. (67.3%) and Mishra et al. (20.7%)[8, 20].

Acceptance increased when the respondent was the sole decision-maker or counseled during a cesarean section. Anecdotally, it is seen that it is easy to guide and convince about the benefits of PPIUCD usage the educated women who have a say in the decision-making process. However, the decision-making among uneducated women is primarily influenced by their family members and guided by societal norms—most of the PPIUCD acceptors who were counseled decided after discussing it with their husbands. The involvement of husbands during the counseling session increases the chances of acceptance of PPIUCD as contraception[20–22]. However, men must be engaged in counseling through tailor-made approaches[23]. We also counseled the women adequately about the benefits of PPIUCD during their intrapartum period, which essentially convinced them to choose PPIUCD as a family planning method, similar to observations made in previous studies [24, 25]. Women undergoing cesarean section showed a higher acceptance rate of PPIUCD, similar to other studies from India and abroad[7, 15, 26, 27]. This can be attributed to fear of post-cesarean conception over the scarred uterus. Previous systematic reviews have reported that post-placental placements during cesarean delivery are associated with lower expulsion rates than post-placental vaginal insertions without increasing rates of postoperative complications.

[28, 29] Satisfaction from the counseling services is also a significant determinant of acceptance and long-term retention. As per WHO, the role of counseling cannot be ignored as it supports a woman and her partner in choosing the method of family planning that best suits them. It also involves them in making an informed decision and addresses any concerns with the selected contraceptive method. Thus, if a woman can make an informed choice about any contraceptive method, it increases her acceptance, satisfaction, and usage [30].

The most common reasons for PPIUCD acceptance that emerged in our study align with the existing literature [16, 24, 31–33]. Immediate postpartum period is the appropriate time to begin contraception as women are highly motivated and not known to be pregnant. There is no need for additional hospital visits for insertion, which also has the advantage of being convenient for both women and health care providers. We observed that the most common reasons for declining PPIUCD were resistance from the family members, notably the husband, insufficient knowledge about PPIUCD, preference for other less invasive methods of contraception, and fear of side effects, similar to other studies [16, 24, 31–33]. Nigam A et al. have also pinpointed ineffective counseling as a crucial factor for refusal [34]. In our hospital, women were not accompanied by their partners during the antenatal visits, which deprives male spouses of understanding the PPIUCD benefits. Thus, partner refusal was the most common reason for denying PPIUCD. This again highlights the importance of partner involvement during counseling and decision-making, as stressed by previous studies [21–23].

The most common complications following PPIUCD insertion included AUB, missing thread, and infections, the most common reason for removal. Most of the missing thread complications were seen within six weeks in the women who had intra-cesarean insertion of PPIUCD because, at the time of insertion, there is the practice of leaving the entire length of IUCD string in the uterine cavity and not passing it through the cervical-os which leads to curling up of thread that is not visible at external-os. This may cause apprehension as the missing thread relates to expulsion, malposition, and perforation. However, there was no reported case of uterine perforation. None of the studies retrieved during the literature review has reported uterine perforation after PPIUCD insertion, except in one case report [35].

In the present study, we observed a continuation rate of about 80.6% and a removal rate of 13.9% at six months postpartum. This is similar to the results from previous studies by Ranjana et al. and Sunita Singhal et al. [36, 37]. Cox regression depicted that PPIUCD retention was affected by the type of occupation, religion, and normal vaginal delivery. About one-fifth of our respondents either got their PPIUCD removed, or it was expelled by the end of 6 months. We explored the **reasons for the removal** of PPIUCD. The main reasons for removal included pain, AUB, infection, and family pressure. These factors are in coherence with the existing literature [25, 34, 37–41]. Persistent pain was seen as a critical factor leading to removal. However, two-thirds of our participants perceived no pain during and after PPIUCD insertion, comparable with the study by Kumar S et al. [25]. This can be attributed to the use of the proper insertion technique by skilled health-care workers. Family pressure has been a critical determinant of retention of PPIUCD and a bottleneck for the effective implementation of women’s reproductive rights [8, 39, 40]. We observed a **cumulative expulsion** rate of 5.6% at the end of six months. In previous studies from India, the expulsion rate has been seen between 5 and 10% [8, 11, 25, 42, 43]. Expulsion rate can be minimized if PPIUCD is inserted by a trained health care provider and proper fundal placement by placental forceps. When the IUCD is inserted immediately after the third stage of labor, expulsion rates at six months range between 31 and 41% per a WHO multi-centric trial and from 12 to 22% in a Family Health International multicentre trial [4]. Insertion 1–7 days after delivery results in even higher expulsion rates and may vary between 5 and 10% [37, 41, 44]. Findings in the present study that expulsion is more common in post-placental insertion than intra-cesarean insertion is supported by many studies worldwide [8].

There are certain limitations in the present study that should be acknowledged. Being a time-bound study, we could not do a long-term follow-up of the participants and assess the actual impact of PPIUCD. Excessive bleeding may contribute to high removal rates of PPIUCD after six months of delivery, which warrants further long-term follow-up as the same was not possible due to feasibility issues during the study. We also could not record the patterns of bleeding that prompted women to request IUD removal, which can be done in future studies. We could not rate the quality of PPIUCD counseling services and compare it with existing literature regarding the acceptance rates of PPIUCD. Our study’s follow-up loss was high (15%), similar to observations made by previous researchers field[45]. The loss of patients to follow-up is a significant problem in low and middle-income countries like India. Patients with poor education, low socio-economic status, and poor access to health care facilities tend not to give adequate importance to health-related issues. Lastly, this study was conducted in a health facility; hence the findings might not adequately reflect the entire population.

There is a pertinent policy implication of this study. Our study highlights the need to focus on the continuation rates rather than just the initial acceptance rate of PPIUCD. In the long run, the number of women years counted free of unwanted pregnancy has long-term effects on decreased Total fertility rates and population stabilization. Even if the lower proportion of women accept PPIUCD as a contraceptive method of choice but continue to use it for a longer time, it can trade off the need for a more significant number of women initially to accept PPIUCD. Previous reports have stated that health professionals discourage IUD removals within a year of placement regularly and downplayed or offer to treat side effects instead of giving patients a choice to have their IUD removed immediately [46]. Forcing women to continue using contraceptive methods against their will can diminish trust in the health personnel and patient satisfaction with the technique. Also, such coercive dynamics are against medical ethics and violate the principles of reproductive rights, emphasizing that health professionals should respect a woman’s bodily autonomy and decision-making.

## Conclusion

We conclude that PPIUCD is well accepted by the women as a practical family planning method provided it is offered as an informed choice, followed up for the complications, and supported through adequate counseling. Our study highlights various modifiable and non-modifiable patient-related factors that affect acceptance and facilitates long-term retention. These factors should be discussed during the counseling sessions in the ante-natal periods. The spouse’s involvement is equally crucial to reap this highly effective contraceptive’s long-term benefits. Being a tertiary care center, we observed better retention rates that reiterate our emphasis on enhancing the skills of health care personnel through training, elaborate antenatal counseling, and public awareness campaigns to increase the acceptance of PPIUCD. Retention rates increase when women are adequately enquired about the side effects of PPIUCD in the post-partum period, which can be done during home-based post-natal care visits. At the same time, it is also essential to understand that to meet our targets; health workers should prevent engaging in inadvertent coercion, which may hamper the reproductive rights of the women, and dilutes the whole idea of empowering our women for a better future.

## Data Availability

The datasets for the current study are available from the corresponding author upon reasonable request.

## Abbreviations

IUCD: Intra-Uterine Contraceptive Device
PPIUCD: Post-Partum Intrauterine Contraceptive Device
ANC: antenatal care
PROM: Premature Rupture of Membranes
HIV: Human Immunodeficiency Virus
WHO: World Health Organization
SES: Socio-Economic Status
